# Implementing new routines in adult mental health care to identify and support children of mentally ill parents

**DOI:** 10.1186/1472-6963-14-58

**Published:** 2014-02-07

**Authors:** Camilla Lauritzen, Charlotte Reedtz, Karin TM Van Doesum, Monica Martinussen

**Affiliations:** 1 Regional Centre for Child and Youth Mental Health & Child Welfare, University of Tromsø, Tromsø, Norway; 2 Behavioural Science Institute, Radboud University Nijmegen and Mindfit, Mental Health Centre, Deventer, The Netherlands

**Keywords:** Implementation, Changed clinical practice, Children of mentally ill, Parental mental health

## Abstract

**Background:**

Mental health problems are often transmitted from one generation to the next. This knowledge has led to changes in Norwegian legislation, making it mandatory to assess whether or not patients have children, and to provide necessary support for the children of mentally ill patients. The main purpose of this study was to evaluate the process of implementing new routines in adult mental health services to identify and support children of mentally ill parents.

**Methods:**

The design was a pre-test post-test study. The sample (*N* = 219 at pre-test and *N* = 185 at post-test) included mental health professionals in the largest hospital in the region, who responded to a web-based survey on the routines of the services, attitudes within the workforce capacity, worker’s knowledge on the impact of parental mental illness on children, knowledge on legislation concerning children of patients, and demographic variables.

**Results:**

The results of this study indicated that some changes are taking place in clinical practice in terms of increased identification of children. Adult mental health services providing support for the children was however not fully implemented as a new practice.

**Conclusion:**

The main finding in this study is that the *identification* frequency had increased significantly according to self-reported data since the Family Assessment Form was implemented. The increase in self-reported identification behavior is however taking place very slowly. Three years after the legislation was changed to making it mandatory to assess whether or not patients have children, it was still not fully incorporated in the routines of the entire workforce. In terms of *support* for the families affected by parental mental illness, the changes are not yet significant.

## Background

Children who have parents with a mental illness are at risk for developing psychological problems themselves [1,2]. Parental mental illness is considered one of the most potent risk-factors for the development of psychopathology in the general population [3,4]. However, consequences of parental mental illness is also considered a modifiable risk-factor, in terms of the malleability of parenting practices [5]. In order to prevent the transmission of mental health problems from one generation to the next, it is important to integrate a focus on children when parents receive treatment for mental health problems.

Norwegian health legislation was altered in 2010 due to unsystematic and insufficient services available when parents are mentally ill. The purpose was to increase the identification and follow-up of the children of patients within adult mental health services [6,7]. However, studies have shown that implementing a change of practice in adult mental health care to identify and support children of mentally ill patients is challenging [8-11]. One of the most critical issues in mental health services research is the gap between what is generally known about effective treatment and what is provided to consumers in routine care [12]. The risk of trans-generational transmission of socio-emotional problems and psychopathology as well as the benefits of early intervention has been thoroughly documented the last decade, [13-16]. However, a change of practice in the mental health care system is not easy to accomplish. There are several explanations in the literature as to why changing clinical practice is difficult. The difficulties the practitioners report can be summed up as follows: “Not mine, not trained, too busy, too risky” [17,18]. In the baseline-analyses of the current study [10,11], several challenges were discovered. These were related to lack of ownership to the issue, lack of training, lack of time, insecurity and lack of knowledge. These may be referred to as lacking readiness to change. Readiness to change refers to organizational members’ shared intention to implement a change and a shared belief in their capability to do so [19]. There are three key determinants related to organizational readiness to change and implementation capability: task demands, resource availability and situational factors [19]. Another central issue is whether or not the workforce considers that there is a need for improvement of clinical practice. Previous research has consistently found what has been called a “positive self-assessment bias” [20]. This implies an overly positive assessment of personal performance among mental health professionals, as well as in a wide variety of other occupations. In a study of therapists’ self-perceptions [21] of their own clinical skills and performance levels, compared to others in their profession, 25% viewed their skill level to be at the 90^th^ percentile when compared with others, and none viewed their skills as below average. If this reflects the reality, there would neither be need nor room for improvement among the clinicians regarding identification of and support for children of mentally ill patients, because their current practice is perceived as sufficient.

To deal with the challenges related to changing clinical practice, a systematic strategy to form the basis of the activities in such processes is needed. To incorporate implementation theory when new routines are planned and guidelines developed is one way to systematize such a process. Implementation is defined as an active and planned process of mainstreaming an innovation within an organization takes place [22]. The innovation in this context is a changed clinical practice so that children of mentally ill patients are being identified and offered support. In addition to increased awareness of the pitfalls and hindering factors documented by others, an implementation theory approach provides a framework for rigging implementation efforts in an adequate way. It is important to recognize the fact that innovations do not come about by themselves [23]. The different stages in the process of implementation are described within the theoretical framework we chose in this project [24]. This approach sees implementation as a process with several central stages as opposed to a single event.

**Figure 1:**
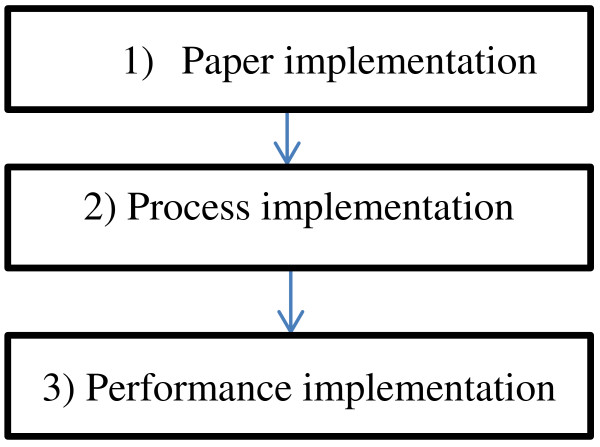
**Degrees of implementation based on Fixsen et al., [**[24]**].**

According to a synthesis of the implementation literature [24], there are three different degrees of implementation; paper-, process- and performance implementation. In order to change clinical practice to safeguard children of mentally ill parents, the initial strategy of the health authorities has been to pass new legislation. Relying on new policies to generate a new way of addressing issues in practice is referred to as *paper implementation*, and according to the literature [24] this rarely leads to innovations in practice that will benefit the patients. Figure 1 provides an overview of the degrees of implementation according to the framework the present study is based on. In Northern Norway the Regional Health Authorities organized a strategy of implementing the new legislation, which involved key groups of the workforce appointed as “child responsible staff”. They were given lectures, information about the risk-factors related to parental mental illness and they were urged to start identifying patients’ children. This strategy may be seen as *process implementation*, where there were several activities related to the topic. However, the content was not systematically related to the new practice in terms of tools or detailed procedures. The activities mainly involved courses about children of mentally ill parents as a high-risk group, and subsequently little training in how to translate the new legislation into practice among the total workforce. Researchers at Tromsø University had prior to the changed legislation developed a project called the BAP-study, in collaboration with the largest hospital in Northern Norway. The BAP-study was designed to provide the “deepest” degree of implementation according to the chosen model; a *performance implementation* approach to the field of practice. This involved developing procedures with functional tools to be adopted in clinical practice and to guide the development of new skills. A key-aspect when aiming to change clinical practice is to enhance the workforce capacity so that the workers are enabled to put into action a practice that includes the children of patients [25]. In order to successfully develop new clinical practice, knowledge on its own is rarely [25,26]. The adult mental health workforce is generally trained to focus on symptoms and treatment, and traditionally do not have a focus on involving the children of the patients. Therefore, to reach the degree of *performance* implementation the new practice must contain tools and well described procedures.

The project included implementation of two interventions; Family Assessment Form, and Child Talks. The Family Assessment Form is an intervention for practitioners to increase identification of patients who are also parents and their children. The intervention is a tool in which the patient is asked to provide information about their children (do you have children, how many, do you have custody etc.). This assessment is made mandatory by the new legislation in 2010. The Family Assessment Form was generally implemented in the admission of new patients, and at the end of this conversation the health care workers were to offer the intervention Child Talks to the parent [27]. *Child Talks* is an intervention designed to provide support for parents and children within the participating hospital. Child Talks is a health-promoting and preventive intervention where the mental health worker talks with the family about the situation of the children and their needs [28]. Child Talks was designed to reduce the risk for the children of patients to develop psychological problems by allowing health care workers to talk to the parent and the children about the situation. Child Talks consists of two to three conversations with the parent and the family, and the focus is information about the disease and possible consequences for the children. Additionally, the aim of this intervention is to provide support for the family and assist them in locating additional services when needed. The organization Adults for Children (VFB) is the program owner in Norway, and has adopted and translated this intervention. VFB is a non-governmental organization (NGO) working to promote good mental health in children and adolescents.

### Aims of the study

This study is part of a large implementation study that evaluates the process and effects of implementing new routines and interventions within adult mental health services in the largest hospital in Northern Norway. The purpose of the present study was to investigate: a) to what extent health personnel had changed their practice in terms of identification of patients’ children, b) had changed their practice in terms of supporting patients’ children, c) had changed their attitude regarding the need to change clinical practice in terms of identification and support for patients’ children, d) if the implementation of new routines had led to changes within the workforce in terms of beliefs, attitudes, knowledge and, concerns about the patient-therapist relationship being interrupted by bringing up a focus on the patient’s children, and e) if there were differences in Workforce Beliefs, Knowledge, Concerns and Attitudes between staff that had implemented the Child Talks and staff that had not.

## Methods

### Participants

The participants of this study are staff and leaders at the largest hospital in Northern Norway. The total workforce was asked to answer a baseline questionnaire prior to the initial process of implementing new routines. A total of 219 individuals responded, representing a response rate of 50%. The respondents were 76% women, and the majority was between 30 to 50 years old.

At post measures the same questionnaire was sent out to the same group of staff and leaders. However, due to the anonymity of the respondents, the samples at pre and post are independent. A total of 185 individuals responded at post-test, representing a response rate of 40. 5%. The respondents were 67% women, and the majority was between 30 to 50 years old. Detailed demographic information is displayed in Table 1.

**Table 1:** Descriptive statistics for demographic and practice-related variables

	**Pre *(N = 219)***		**Post *(N = 185)***	
Gender	Women	76%	76%	73%
	Men	24%	Men	27%
Age				
	<30 years	11%	<30 years	6%
	31-40 years	26%	31-40 years	21%
	41-50 years	26%	41-50 years	35%
	51-60 years	27%	51-60 years	28%
	>60	10%	>60	10%
Level of Education				
	High	15%	High	15%
	Medium	45%	Medium	33%
	Low	40%	Low	51%

### Measures

#### Demographic and work characteristics

Personal demographic variables included age, gender and education, in addition to single items on work characteristics such as leadership responsibilities and current position. Education was divided into three groups; low (under bachelor level), medium (bachelor), and high (master/equivalent or higher).

#### Routines for identification

One question was included: “Do you identify children of patients?”, and participants answered with *yes* or *no*.

#### Beliefs regarding effects of implementing an intervention

The beliefs of the new routines leading to positive outcomes for the children were assessed using a scale consisting of four items and the scale was computed as a mean score of the items. The Chronbach’s alpha for this scale was .86. Items included questions about the expected outcome for patients and children. For instance: “I believe conversations with and about children may contribute to the improvement of the life situation for children of mentally ill parents”. Four items were answered on a five point Likert-scale from “To a very little extent” (1) to “To a very large extent” (5).

#### Knowledge

Materials for assessing status quo in regular practice and changes in clinical practice were based on the *Keeping Families and Children in Mind Online Resource – Evaluation, pre-training survey*. This measure has been evaluated in Australia, and was reported to be a useful tool in this context [29]. With permission from the authors, the questionnaire was adapted to the Norwegian context to assess regular practice in the organization for dealing with children of mentally ill parents. Items included questions on level of knowledge about children and the new legislation. The items were answered on a five point Likert-scale from “To a very little extent” (1) to “to a very large extent” (5). An example of these questions was: “To what extent would you say you have knowledge about the consequences of mental illness for the parenting role?” The number of items was ten, and factor analysis was used to reduce the number of items. The suitability of data was assessed to be good, with a KMO-value of .85 and a significant Bartlett’s test. Principal Components Analysis (PCA) with Varimax rotation indicated two factors. One group of items focused on general knowledge about children and parenting and a second group of items addressed specific knowledge about legislation and guidelines. Two scales were computed based on mean scores of the respective items and were named Knowledge Legislation and General Knowledge. The computed Cronbach’s Alpha was .73 for the Knowledge Legislation scale and .90 for the General Knowledge scale.

#### Attitudes towards implementing new routines in mental health care for adults to identify and follow-up children of patients

The scale included eleven items. E.g. “Health personnel should identify whether or not patients have children”. The items were rated on a five-point scale, from “To a very little extent” (1) to “To a very large extent” (5). The 11 items of the Attitudes scale were subjected to Principal Components Analysis (PCA). Prior to performing PCA, the suitability of data for factor analysis was assessed. The KMO-value was .85, exceeding the recommended value of .6 [30]. Bartlett’s test of sphericity was significant, supporting the factorability of the correlation matrix. Varimax rotation was used, and two components were discovered. The interpretation of two components was consistent with previous research on the attitudes scale [11]. Positive attitudes toward a focus on the patient’s children in the treatment of mentally ill parents loaded strongly on component 1. Negative attitudes concerning the interruption of treatment and the interruption of the parenting role loaded into component 2. Two scales were computed based on mean scores of the items of the respective components. The scales were named Positive Attitudes and Concerns respectively. Reliability analyses were conducted o and the computed alpha for Positive Attitudes was .91, and .76 for Concerns.

#### Family conversations

Experience with family conversations was assessed using one item: “To what extent do you have experience with family conversations?” The item was rated on a five-point scale, from “To a very little extent” (1) to “To a very large extent” (5).

#### Quality assessment

One item tapped into quality self-assessment: “To what extent would you say that the services your clinic offer to children of mentally ill parents are adequate?” The item was rated on a five-point scale, from “To a very little extent” (1) to “To a very large extent” (5).

### Procedure

As a result of the new legislation which made it mandatory to identify and follow-up children of mentally ill parents [7], all hospitals in the northern region of Norway received information in form of a directive [6]. This directive describes the new legislation and provides detailed comments on the new policy the health personnel are expected to adopt. The new policy entails that health personnel are to identify minor children, as well as provide information and support for children of patients with a mental illness. The directive does not however provide the health care services with systematic routines or guidelines in terms of how the new practice is to be operationalized. In addition, the health personnel were given one-day courses that encompassed information about the new policies and procedures that were expected to be implemented due to the new legislation [6]. However, the way the hospitals were to adopt and implement the new operating routines was not standardized. To a large extent each hospital had to make decisions about procedures themselves. In collaboration with the largest university hospital in northern Norway, the BAP-project was therefore designed to systematize procedures in such a way that the implementation of new routines was evaluated continuously. The evaluation of the implementation process is a part of a 10-year longitudinal (pre-post-one year follow-up) study, and the new practice is use of the interventions *Family Assessment Form* and *Child Talks* in the hospital.

**Figure 2:**
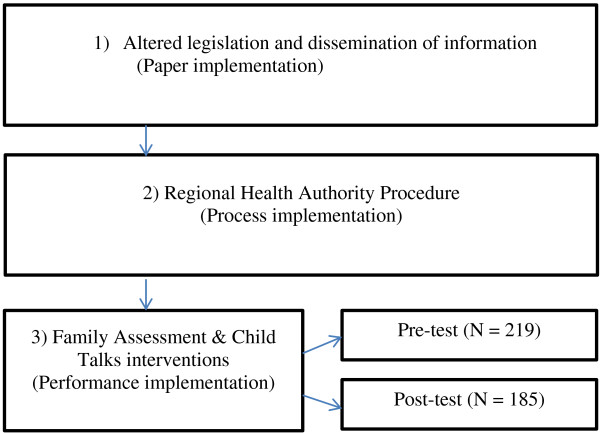
Stages of implementation in the BAP-study.

Electronic survey questionnaires were used for both measurement times (Quest Back), and the questionnaires were completed anonymously. To illustrate at which stages we collect data in our study, we have inserted the times of measurement into the implementation model that was our framework (T1-T2). T1 represent the pre-test group and T2 represent the post- test group. T3-data will be gathered in a follow-up study of the implementation process, when the changes that were implemented are at full operation [Figure 2].

The study was approved by the Data Protection Supervisor at the University Hospital of Northern Norway, and was conducted in line with the Helsinki Declaration of Ethical Principles for Medical Research Involving Human Subjects published by the World Medical Association [31].

### Data analyses

The data was exported from Quest Back to SPSS. All statistical analyses were performed with SPSS (Version 19). Descriptive analyses were used to explore the demographic details of the groups. Independent samples t-test was used to test the differences between the groups. Cohen’s *d* was calculated to express effect sizes [32]. According to Cohen’s criteria, Cohen’s *d* = .80 is considered a large effect, *d* = .50 is considered a medium effect and *d* = .30 is considered a small effect [32]. Pairwise missing was used for descriptive analyses and t-tests. In general, the data set had few missing values.

## Results

Self-report data showed a significant increase in self-reported identification behaviour from pre to post in the total workforce. Compared to pre, where only 44% reported that they were using the Family Assessment Form to identify children, 65% used the Family Assessment Form at post test. The difference was significant (Chi^2^ = 18.6, *p* < .05).

To investigate whether or not the workforce had changed their practice in terms of providing support for the children, we tested for increased experience with family conversations. There were no significant changes in terms of family conversations three years after the new legislation made this mandatory (see Table 2).

**Table 2:** Independent samples t-tests of differences in outcome variables between pre-test and post-test

	**Pre-test**		**Post-test**		***t***	**Cohen’s *d***
	**(*N*** **= 211–219)**		**(*N*** **= 181–185)**			
	*M*	*SD*	*M*	*SD*		
Beliefs	4.25	0.65	4.04	4.04	3.39**	0.32
General Knowledge	3.29	0.71	2.54	0.71	9.01***	1.06
Legislation Knowledge	2.99	0.75	2.90	0.71	1.92	0.12
Concerns	2.38	0.74	2.46	0.77	-1.01	-0.49
Positive Attitudes	4.22	1.08	4.44	0.58	-2.44*	-0.25
Family Conversations	3.24	1.17	3.08	1.04	1.47	0.14
Quality Assessment	2.62	0.91	2.23	0.75	4.64**	0.47

*Note.* *p < .05. **p < .01. ***p < .001 (two-tailed test).

All hospital wards are obligated to appoint a member of the staff to be a “child responsible person” due to the altered legislation. A child responsible person has a particular responsibility to ensure that their ward is professionally equipped and updated in terms of providing support for families. This does not mean however that they have the sole responsibility for the children of patients within the wards. The child responsible persons are appointed by the ward managers, and we wanted to test if they were significantly different from the rest of the workforce in terms of providing support for children of patients. We tested if the child contacts reported a higher number of family conversations (Child Talks). There were no significant differences between the child contacts and the rest of the staff in terms of providing support for children of patients as a part of the Child Talks intervention. At post-test, 31% of the total staff reported that they had offered Child Talks to patients who were parents, and 25% reported that they had delivered the intervention to families. This means that ¾ of the personnel had not started to use the Child Talks intervention.

Additionally, we asked the workers to assess the quality of the services they provided for patients who are parents and their children. At pre-test a total of 91% reported that the services provided by their clinic were adequate. At post-test, this number had decreased to 82%. The self-assessment of the Quality of Services had decreased significantly from a mean score of 2.62 (*SD* = 0.91) to a mean score of 2.23 (*SD* = 0.75). The difference in terms of Cohen’s *d* was 0.47, representing a small to medium effect according to Cohen’s criteria [32]. Results are displayed in Table 2.

We also wanted to investigate whether or not the workforce scored significantly different from pre-test to post-test on the core variables Knowledge, Attitudes, Concerns and Beliefs. Detailed results are displayed in Table 2. There were no significant differences in Concerns between the pre-test and the post-test groups, indicating that the implementation of new routines had not lead to the workforce being more or less concerned about a child focus interrupting the patient-therapist relationship. Furthermore, Positive Attitudes toward incorporating routines to identify and support patients’ children had increased. The effect size in terms of Cohen’s *d* was 0.25; representing a small effect. There were significant differences in terms of Knowledge and Beliefs between the pre- and post-group. The post-test group scored lower on both variables. Those who responded at pre-test reported more positive beliefs for good outcomes as a result of the new interventions than those who responded at post. Additionally, the pre-test group stated they had more knowledge about children and risk factors of parental mental illness than the post group. We also tested for differences between the staff who reported they had started to use the Child Talks intervention and the staff who had not. The only significant difference we found was in Beliefs. Results are displayed in Table 3. The staff that had started running the Child Talks intervention had higher expectation to the outcomes of intervening to support children of parents who have a mental illness. The difference was *d* = 0.61, which indicates a medium effect according to Cohen’s criteria [32].

**Table 3:** Independent samples t-tests of differences in expectations, knowledge, concerns and attitudes between staff that have implemented the child talks and staff that has not

	**Have used Child Talks**		**Have not used Child Talks**			
	**(*n*** **= 46)**		**(*n*** **= 137)**			
Scales	*M*	*SD*	*M*	*SD*	*t*	Cohen’s *d*
Beliefs	4.30	0.48	3.94	0.68	3.36**	0.61
General Knowledge	2.51	0.87	2.56	0.64	-0.36	-0.07
Legislation Knowledge	2.88	0.81	2.91	0.67	-0.21	-0.04
Concerns	2.28	0.81	2.53	0.73	-1.92	-0.32
Positive Attitudes	4.39	0.70	4.45	0.53	-0.62	-0.10

*Note.* **p < .01. (two-tailed test).

## Discussion

This project was designed to facilitate change of clinical practice by implementing two interventions in the participating hospital. We evaluated to what degree clinical practice was changing in order to identify and offer patients’ children support. The results indicate that some changes in clinical practice have started to materialize.

The main finding in this study is that the identification frequency has increased significantly according to self-reported data since the Family Assessment Form was implemented. The number of mental health workers who report they have started to systematically identify whether or not patients have children is higher at pre-test than at post-test. Even though we see an increase in self-reported identification behavior, the change is coming along very slowly. Three years after the legislation was changed to making it mandatory to assess whether or not patients have children, it is still not fully incorporated in the routines of the entire workforce. In spite of the implementation strategy, the results show that there still is a lack of awareness in adult mental health services that their clients may have children and that there is still barriers to overcome [17,26]. One explanation for this may be that the majority of the workers already had the notion that they were delivering adequate services for the patients who were parents, and the reason to change practice was thereby less impending. Previous research has documented that a vast majority of health care workers assess the quality of the services they provide as high [20,21]. This would imply that the need for changes in practitioners’ behavior is low. This interpretation of our results is in line with the American studies conducted by Lambert and colleagues on the issue of enhancing treatment effects [20,21]. Nevertheless, this study detected some changes in attitudes among the mental health care workers regarding self-assessed quality of the services they provide. They regarded the quality of the services to be less appropriate at post-test, possibly indicating a changed awareness about what is actually needed to provide sufficient support for children. According to Walfish and colleagues [21], the self-assessment bias generally causes practitioners to rate their own practice higher than it should be rated. We believe that the positive self-assessment bias may have caused the workforce at pre-test to overestimate the quality of the existing services on these matters. However, according to Lambert [20], providing the health care workers with information about limitations in their practice has been proven to counteract the failure in accurately predicting service outcomes and quality [20]. We believe the implementation strategy may have sensitized the workforce in terms of shortcomings in the services provided, and that this may have caused their self-assessment of quality to go down. Rather than evaluating the current practice as good-enough, the workers may have come to the realization that the services offered were insufficient. This may indicate an awareness amongst the workers that what they are doing is not sufficient.

Even though the workforce may have developed more realistic perceptions about the quality of the services, the support for children has not been fully incorporated in clinical practice. In fact, the number of workers who say they have experiences with family conversations has decreased from pre-test to post-test (see Table 2). We believe this may be due to family conversations having different connotations to the staff at post-implementation compared to at pre-test. At pre-test we had not started to implement child talks, and therefore the reported experiences with family conversations were more general and not linked to the specific intervention to be implemented. Even though there is a significant increase in self-reported identification of children, the implementation of the intervention Child Talks still seems very challenging for the mental health care workers. Only one out of four reported that they had started using the intervention Child Talks with one or more families. These results may be seen as an indication of lacking readiness to change, and suggests that more work need to be done to create such readiness within the workforce. Activities to make the task demands comprehensible, to allocate adequate resources and to operationalize the situational factors that are hindering the capability to implement change, may create increased readiness to change within the workforce [19]. Readiness to change is reflected in the beliefs, attitudes and intentions of organizational members in addition to the organization’s capacity to make those changes. According to Weiner, the workforce is more likely to initiate change, exert greater effort, exhibit greater persistence and cooperate better when organizational readiness for change is high [19]. These are all factors which need attention in this longitudinal project. The finding that ¾ of the workforce has yet to start using the Child Talks intervention, suggests that there is much work to be done in both the management and workforce of the hospital. A wider implication of the results may be that other Norwegian hospitals, without specific implementation projects on these matters, may not be able to change practice according to the new laws.

In addition to studying the actual behavior on identification in the clinic, we also wanted to see if the groups were different on the core variables Knowledge, Concerns, Attitudes and Beliefs after the initial implementation activities were completed. The post-group scored higher on positive attitudes, which can be expected after receiving systematic training in how psychopathology can be prevented from being transmitted from one generation to the next [1,15]. The emphasis on the importance of identifying and supporting the children seems to have led to more positive attitudes within the workforce. Nevertheless, when you study the scores in detail, the difference between pre and post on these core variables may not be of much clinical importance after all. Even though the difference is statistically significant, both numbers refer to very high ratings of positive attitudes, representing a possible ceiling-effect. In terms of concerns related to whether or not bringing a child- and parenting focus into the treatment may interrupt the patient-therapist relationship, there was no change from pre-test to post-test. If therapists had experiences which caused them to be more concerned about the therapeutic alliance, we assume they would have less positive attitudes about including a child and parenting perspective. Furthermore, if concerns had gone up, a logical consequence would be that the resistance to including the new routines also would be increasing, which was not the case with our data. Therefore, we believe this result can be interpreted as evidence that the workforce readiness to change is increasing.

What surprised us most was the change in self-reported knowledge about risk-factors and beliefs of the interventions representing positive outcomes for children, because the scores had actually gone down from pre to post. One possible explanation for this may be in line with the statement “the more I learn, the less I realize I know” (Socrates). Having participated in courses about the risk-factors and consequences of parental mental illness may have sensitized the workforce on the complexity of the topic, and may have given them experiences with many of the implementation challenges in their work to adapt the new routines into practice. The Child Talk Intervention is now in their “hands” and no longer a theoretical intervention, and due to several factors making it difficult for them to fully put it into use, they may therefore have more moderate expectations to it being effective. We believe that this is due to their current realistic viewpoint, as opposed to their initial and more positive viewpoint, and that this is the reason that they evaluate their existing practice less positively. The decrease in self-reported expectations and knowledge may be interpreted as an indication that the workforce have realized there are several barriers to overcome until it will be possible for them to fully incorporate the new practice in current routines.

It seems that the workforce needs other conditions to implement a clinical practice where they identify and support patients’ children. In our view, these conditions are mainly related to adding various resources to the existing clinical practice, such as; time, tools, new routines, training, education and sufficient staff [10]. Additionally, the distribution of responsibility needs to be made clearer as to whom within the workforce should be the ones taking on the new tasks. Instead, the participating clinic has been subject to major financial cuts, and strong prioritizing between many law-enforced activities. This aspect is important to keep in mind when the results of this study are interpreted, because taking care of the patient’s children may not be a prioritized task when priorities have to be made. The treatment of the adult patient in itself will naturally be the main focus, and additional tasks such as providing support for the children are likely to be given less attention. To achieve a broader view on how to treat mental illness that includes a focus on the parenting role, we believe this perspective needs to be included in the education of mental health workers, i.e., in the syllabus of the undergraduate level of the education of psychologists, psychiatric nurses, psychiatrists etc.

Still, it is an indisputable finding that mental illness is transferred from one generation to the next [14,15]. A prevention focus in the treatment of mentally ill parents may reduce the transmission of mental illness from one generation to the next. We believe, that given patience and the adequate tools, the capacity within the workforce can be developed to fully incorporate this perspective. Feasibility is however crucial. Implementation strategies must include clear allocation of resources, clear definitions of what the new practice consists of in terms of tasks and responsibilities, and the time-aspect of implementation work must be taken properly into account. According to Fixsen and colleagues [24], evaluation of newly implemented programs may result in poor outcomes, not because a program is ineffective, but because the results at the implementation site were assessed before the program was completely implemented and fully operational. Changes in organizational culture and skill levels require time to mature, and the resistance to change needs enough time to be dealt with [24,33]. The BAP-study may not have been running long enough for all the changes to have materialized, and clearly, more work needs to be done on strengthening readiness to change and improve conditions to make changes possible. This is a prerequisite in order for the implementation to move into full operation.

In the continuation of the BAP-study, there are several steps to take in order to encourage the process of implementing new practice. Firstly, it might be fruitful to initiate meetings with management and to involve the managers in the process of fostering readiness to change. Allocation of staff resources, time and arenas are factors in which management needs to actively be involved in order to encourage the implementation process. Secondly, we have initiated booster sessions in terms of training and courses. We also believe that the staff responsible for internal courses may need additional training. In the training of trainers, it might be a good idea to include more training on how to supervise colleagues in carrying out the interventions. Another idea may be to create arenas (or take advantage of existing arenas) to discuss actual cases where patients are parents. Finally, it might be a good idea to turn to the education of mental health workers (e.g. nurses, psychologists, doctors) in trying to stimulate the inclusion of the child and family perspectives in curriculums.

### Study limitations

The study relied on self-report measures for attitudes, knowledge, as well as current identification practice. To make up for this limitation, objective measures in terms of journal data to assess the number of children of mentally ill should be investigated. Another limitation is the relatively modest response rate of this study (50% at pre-test and 40% at post-test). This may bias the results if the decision to participate is related to worker attitudes, e.g., that those who are already positive about involving the children of their patients are more likely to participate. One consequence of this may be that this article presents the attitudes within the workforce as more positive than what they really are. Future studies should also include other explanatory variables to get a bigger picture of the hindering factors related to involving the children of patients who are parents. This may include both individual characteristic as well as organizational variables.

Future studies should also include interviews with the personnel to further explore implementation barriers we have yet to discover. We have therefore initiated a study where key-personnel are being interviewed.

## Conclusion

Systematic implementation strategies to change clinical practice seem to be working, but the changes are coming along very slowly. Implementation work in general consists of inert processes, and strategies must be long-term in order to succeed. In conclusion, the workforce as a whole is moving toward a more open and positive approach regarding the adoption of the new routines. More extensive work to enhance readiness to change and identify barriers is needed to move the implemented interventions to full operation.

## Competing interests

The authors’ declare no competing interests.

## Authors’ contributions

All authors participated in the design of this study. CR and CL obtained funding for the study. CL drafted the manuscript and conducted the analyses. All authors contributed to writing the manuscript, and all authors read and approved the manuscript prior to submission.

## Pre-publication history

The pre-publication history for this paper can be accessed here:

http://www.biomedcentral.com/1472-6963/14/58/prepub
